# Supplementary material for: Universal Computational Super-Resolution Framework for Imaging of Quantum Dots
Source: ACS Nanosci Au. 2026 Aug 5;6(5):842–9. doi: 10.1021/acsnanoscienceau.5c00198 (PMC13693260; doi:10.1021/acsnanoscienceau.5c00198)
Supplement: Supplementary file 1 [file ng5c00198_si_001.pdf]

Universal computational super-resolution framework for imaging of quantum dots

DOMINIK VAŠINKA,[1,†] JAEWON LEE,[2,†] CHARLIE STALKER,[2] VICTOR MITRYAKHIN,[3] IVAN SOLOVEV,[3] SVEN STEPHAN,[3,4] SVEN HÖFLING,[5] FALK EILENBERGER,[6,7,8] SETH ARIEL TONGAY,[9] CHRISTIAN SCHNEIDER,[3] MIROSLAV JEŽEK,[1] AND ANA PREDOJEVIĆ[2,*]

[1]*Department of Optics, Faculty of Science, Palacký University, 17. listopadu 12, 77900 Olomouc, Czechia*
[2]*Department of Physics, Stockholm University, 10691 Stockholm, Sweden*
[3]*Institut of Physics, University of Oldenburg, D-26129 Oldenburg, Germany*
[4]*University of Applied Sciences Emden/Leer, 26723 Emden, Germany*
[5]*Technische Physik, Physikalisches Institut and Würzburg-Dresden Cluster of Excellence ct.qmat, Universität Würzburg, Am Hubland, D-97074 Würzburg, Germany*
[6]*Institute of Applied Physics, Abbe Center of Photonics, Friedrich Schiller University Jena, 07743 Jena, Germany*
[7]*Fraunhofer-Institute for Applied Optics and Precision Engineering IOF, 07743 Jena, Germany*
[8]*Max-Planck-School of Photonics, 07743 Jena, Germany*
[9]*Materials Science and Engineering, School for Engineering of Matter, Transport, and Energy, Arizona State University, Tempe, Arizona 85287, United States*
[*]*ana.predojevic@fysik.su.se*
[†]*These authors contributed equally.*

Supplementary Information

Beyond the demonstrated reconstructions of quantum dot samples, we further analyzed the generalization capabilities of the model. The results presented in the main text already demonstrate its ability to handle an inhomogeneous, structured background, despite not encountering such imperfections during training. Here, we present two additional simulated analyses that extend far beyond the optical conditions covered during training.

Panel (a) of Fig. S1 characterizes the performance on samples with point spread functions that continuously transition along the horizontal axis from low numerical aperture Gaussian profiles to high numerical aperture Airy profiles. Similarly, panel (b) demonstrates performance in the presence of aberrated point spread functions with increasing aberration strength. Namely, we consider astigmatism, coma, defocus, spherical aberration, and trefoil, modeled using full vectorial simulations based on Zernike polynomials [1]. In both panels, model performance is quantified using the structural similarity index measure [2] between the reconstructed images and their corresponding ground truths. As shown, the model exhibits surprisingly strong robustness to these imperfections characterized by the high similarity index, despite never encountering such imaging conditions during training, which further underscores its universality.

It is important to note that this study demonstrates the imaging of quantum dots using a universal, yet small, convolutional neural network trained on simplified simulated data. Both the network architecture and the simulation complexity could be further expanded, potentially resulting in even greater performance and generalization at the cost of additional computational time for training.

References

1. E. Wolf, “Electromagnetic diffraction in optical systems - I. An integral representation of the image field,” Proc. Royal Soc. London. Ser. A. Math. Phys. Sci. **253**, 349–357 (1959).
2. Z. Wang, A. Bovik, H. Sheikh, and E. Simoncelli, “Image quality assessment: from error visibility to structural similarity,” IEEE Trans. on Image Process. **13**, 600–612 (2004).

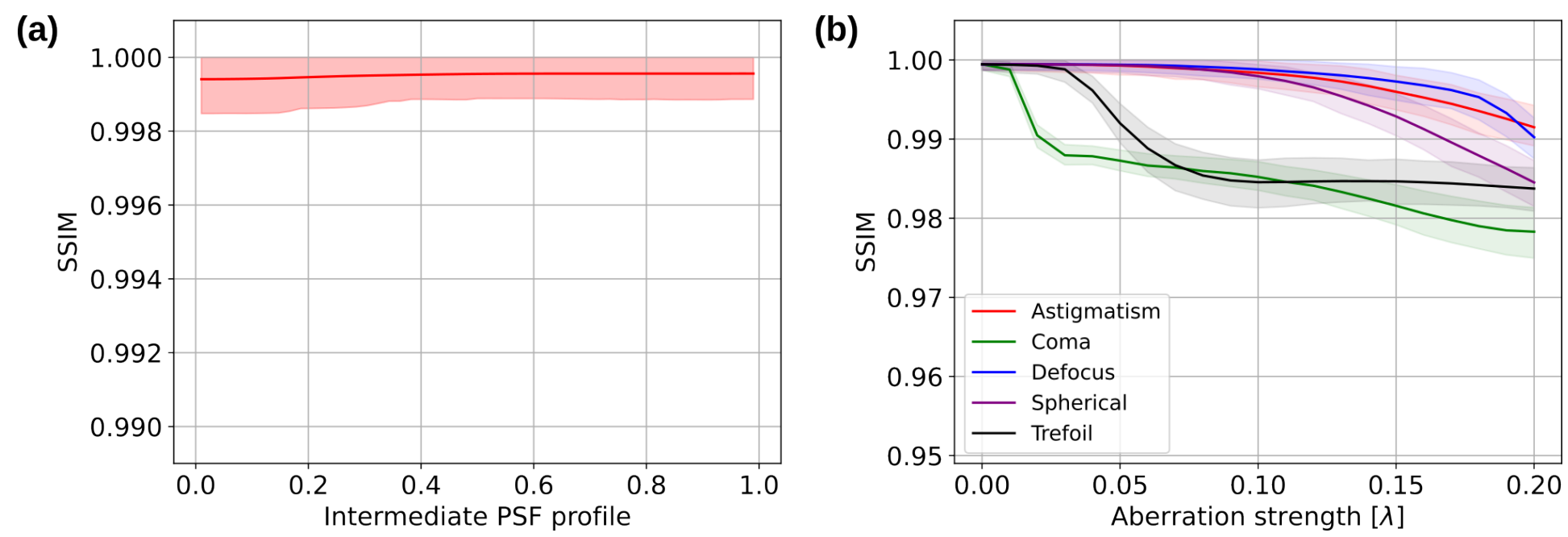

Supplementary Fig. S1. Generalization performance beyond the imaging conditions covered during training. (a) Structural similarity index measure (SSIM) evaluated across a continuous transition from the Gaussian to the Airy point spread function profiles. (b) Robustness to increasing optical aberrations, highlighting the ability to generalize beyond training distributions.
